# Should the use of omeprazole be allowed during equestrian competition?

**DOI:** 10.1111/evj.14129

**Published:** 2024-07-12

**Authors:** Madeleine L. H. Campbell, Benjamin W. Sykes

**Affiliations:** ^1^ School of Veterinary Medicine and Science University of Nottingham Loughborough UK; ^2^ School of Veterinary Sciences Massey University Palmerston North New Zealand

**Keywords:** competition, ethics, horse, omeprazole, social licence, welfare

## Abstract

**Background:**

Prioritising equine welfare, making evidenced‐based policy, and consistent decision‐making across sports are crucial to maintaining the social licence for equestrian sport. Regulations on the use of omeprazole during competition differ; all regulators argue that their rules prioritise welfare. This discrepancy is a matter of concern to the public and equestrian stakeholders.

**Objectives:**

To apply Campbell's Ethical Framework for the use of Horses in Sport to the question: ‘Should the use of omeprazole be allowed during equestrian competition?’

**Study design:**

A desk‐based ethico‐legal study.

**Methods:**

Campbell's Ethical Framework for the Use of Horses in Sport was applied in a stepwise fashion: definition of the ethical question; analysis of the evidence base; consideration of stakeholders' interests; harm:benefit analysis; application of the three central tenets of the framework, and formulation of conclusions and recommendations.

**Results:**

Stakeholders in equine sports have a variety of (frequently conflicting) interests; all of them share an interest in optimising equine welfare. The incidence of EGUS in competition horses is high. Omeprazole is a cornerstone treatment. There are currently discrepancies in regulation about the use of omeprazole during competitions. Recent evidence suggests that withholding omeprazole treatment for two clear days before competition allows the recurrence of squamous EGUS, whereas withholding treatment on the day of competition only does not have that effect.

**Main limitations:**

The current state of scientific knowledge about the use of omeprazole in horses. The analysis did not consider possible health and thus welfare effects of the out‐of‐competition treatment with omeprazole.

**Conclusions:**

Based on recent scientific evidence, if horses are being treated with omeprazole outside of competition then treatment on the day of competition should be permitted on welfare grounds. Revision of regulations around the use of omeprazole during competition by governing bodies is necessary to safeguard the ethical use of horses in sport.

## Resumo

**Contexto:**

Priorizar o bem‐estar equino, elaborar políticas baseadas em evidências e tomar decisões consistentes em todos os esportes são cruciais para manter a licença social para o esporte equestre. As regulamentações sobre o uso de omeprazol durante a competição diferem; todos os reguladores argumentam que suas regras priorizam o bem‐estar. Essa discrepância é motivo de preocupação para o público.

**Objetivos:**

Aplicar o Modelo Ético de Campbell para o Uso de Cavalos em Esportes^1^ à pergunta: ‘Deve o uso de omeprazol ser permitido durante a competição equestre?’

**Desenho Do Estudo:**

Um estudo ético‐legal baseado em pesquisa documental.

**Metodologia:**

O Modelo Ético de Campbell para o Uso de Cavalos em Esportes^1^ foi aplicado de forma gradual: definição da questão ética; análise da base de evidências; consideração dos interesses do público alvo; uma análise de dano:benefício; aplicação dos três princípios centrais do modelo; e formulação de conclusões e recomendações.

**Resultados:**

O público de esporte equino têm uma variedade de interesses (frequentemente conflitantes), enquanto todos compartilham o interesse em otimizar o bem‐estar equino. A incidência de EGUS (Síndrome da Úlcera Gástrica Equina) em cavalos de competição é alta, a qual o omeprazol é um tratamento fundamental. Atualmente, há discrepâncias na regulamentação sobre o uso de omeprazol durante competições. Evidências recentes sugerem que a suspensão do tratamento com omeprazol por 2 dias antes da competição permite a recorrência da EGUS da porção escamosa, enquanto a suspensão do tratamento apenas no dia da competição não tem esse efeito.

**Principais limitações:**

O estado atual do conhecimento científico sobre o uso de omeprazol em cavalos. A análise não considerou possíveis efeitos sobre a saúde e, portanto, sobre o bem‐estar do tratamento com omeprazol fora da competição.

**Conclusões:**

Com base em evidências científicas recentes, se os cavalos estão sendo tratados com omeprazol fora da competição, o tratamento no dia da competição deve ser permitido por razões de bem‐estar. A revisão das regulamentações sobre o uso de omeprazol durante a competição pelos órgãos reguladores é necessária para salvaguardar o uso ético dos cavalos no esporte.

## 1 INTRODUCTION

The continuation of the social licence to use horses in sport is increasingly under threat as wider public attitudes about the use of non‐human animals by humans evolve. There are a number of elements of maintaining that social licence, including transparency and trust.^1^ Key amongst these elements is being able to communicate to the non‐equestrian public the welfare‐based rationale for decision‐making around sports regulations. In this respect, it is helpful not only if decision‐making around rules and regulations is transparent, but also if it is, wherever possible, consistent across sports.

Campbell's previously published ‘Ethical Framework for the Use of Horses in Sport’^2^ provides a tool that can be used across equestrian sports to not only enable but also publicly demonstrate such consistent decision‐making. Using this framework, regulatory decision‐making is informed by the scientific evidence base about the welfare effects of any particular action under policy consideration.

Omeprazole is a proton pump inhibitor (PPI) that is commonly used in veterinary medicine as a treatment for Equine Gastric Ulcer Syndrome (EGUS), including both Equine Squamous Gastric Disease (ESGD) and Equine Glandular Gastric Disease (EGGD). EGUS commonly occurs in horses that are intensively managed: clinical signs may be absent or may include poor appetite, poor body condition, poor performance, behavioural change, and abdominal discomfort.^3^ EGUS affects horses in a wide range of equestrian disciplines including showing, dressage, showjumping, eventing, endurance, western disciplines and racehorses.^3^ Prevalences of up to 100% and 65% for Equine Squamous Gastric Disease and Equine Glandular Gastric Disease, respectively, have been reported in racing Thoroughbreds.^4^, ^5^ PPI drugs reduce the production of acid in the equine stomach by impairing the H^+^, K^+^, ATPase (proton) pump that normally secretes hydrochloric acid. In turn, this provides a favourable environment for healing and alleviates the clinical signs of EGUS that impact negatively upon equine welfare.

The use of omeprazole was identified in the literature as an area of inconsistent regulation across equestrian sports as early as 2013.^6^ The conundrum, and thus regulatory discrepancy, about whether or not to allow the use of omeprazole during competition revolves around a conflict between an ethical imperative to ensure that sport is ‘clean’ and an ethical imperative to alleviate animal suffering when it is possible to do so.

The ethos of ‘clean sport’, that is, sport being conducted with athletes (whether human or non‐human animals) free of drugs relates to the ethical principle of justice.^7^ This approach emphasises the idea of ‘a level playing field’ and, where betting on a sport takes place, of ‘punters’ having confidence that no athlete has an ‘unfair’ advantage over another. It is further argued in the context of equestrian sport that insisting that horses compete free of drugs (with a few exceptions. See, e.g., the British Horseracing Authority's rules on ‘prohibited substances’)^8^ protects equine welfare by disincentivising attempted improvement of performance through pharmaceutical means that might have adverse effects on the short‐ or longer‐term health and welfare of the horses treated. Such arguments form the basis of regulations that do not allow the use of omeprazole during competition.

Conversely, a welfare‐based argument can be made in favour of allowing the use of omeprazole during competition. This argument can be rationalised by the ability to prevent a common disease that is largely induced by management changes associated with training and competing. ESGD and EGGD are both induced and exacerbated by management in that both are prevalent in feral and non‐exercising populations, but the prevalence and severity of both ESGD and EGGD increase with the onset of training.^9^ Given the prevalence of EGUS in horses in training and the apparent difficulty under common management systems in reducing such prevalence through non‐pharmaceutical methods alone,^10^ it can indeed be argued that failure to use such a drug when it is available would be to allow ‘unnecessary suffering’ under the Animal Welfare Act (2006)^11^ through an act of omission.

The fact that regulators of international Thoroughbred racing and the Federation Equestre Internationale (FEI) as a regulator of most international‐standard non‐racing equestrian sports allow the use of omeprazole outside of competition reflects the validity and acceptance of such welfare‐based arguments. However, different regulators have placed differing emphasis on the apparently conflicting ethical imperatives to (i) conduct competition on a ‘clean sport’ basis and (ii) prevent unnecessary suffering through alleviating clinical symptoms where a drug is available to do so. This has led to a position whereby the regulators of Thoroughbred racing do not allow the use of omeprazole during competition whereas the FEI does, with both arguing that their regulation best protects animal welfare. This discrepancy in regulatory approach has continued to be an issue of increasing concern and discussion, for example, at a British Equine Veterinary Association meeting ‘Supporting champions’ themed on medication control held in 2023. Such concern is due both to the direct relevance to equine welfare and because of the damage that inconsistent, and therefore difficult‐to‐explain, regulations might do to the maintenance of the social licence to use horses in sport.

The ethical conundrum around the use of omeprazole in equestrianism was used as one example during initial stakeholder testing of the Ethical Framework for the use of Horses in Sport.^12^ The purpose of that testing was not to reach a conclusion about the use of omeprazole, but rather to test how well stakeholders were able to use the framework tool, and to refine the tool according to stakeholder feedback.^12^ Thus any conclusions that stakeholders reached about the use of omeprazole during the course of that testing were not analysed for publication.

It has hitherto not been clear, prima facie whether or not the use of omeprazole during competition is ethical (and therefore ought to be allowed) because there has been an insufficient evidence base to resolve apparently equally valid and conflicting arguments that (a) equine welfare (as well as sports integrity) is best protected by not allowing the use of drugs during competition and (b) if a drug that can relieve discomfort in competition horses without putting them at exacerbated risk of injury is available it would be detrimental to equine welfare not to use it. Recently, there have been significant and pertinent developments in the scientific understanding of the effects of withdrawal of equine omeprazole. In this article, with particular reference to those recent developments, the authors apply Campbell's Ethical Framework for the Use of Horses in Sport^2^ to the question of whether the use of omeprazole should be allowed during equestrian competition. By using this standardised tool, the authors hope to enable regulators to seek closer alignment of their regulations on this issue, and thereby not only serve equine welfare interests but also increase public confidence in equestrian sport.

## 2 METHODOLOGY

### 2.1 Step 1

Campbell's Ethical Framework for the Use of Horses in Sport^2^ was used to consider the defined question: ‘Is it ethical to allow the use of omeprazole during competition?’ The function of that framework has been previously described in both its original^2^ and a refined^10^ form. Briefly, the tool is centred on three ‘central tenets’ and a stepwise use of evidence and reflection to inform a consensus opinion. The three central tenets are:

- Minimisation of negative welfare and maximisation of positive welfare for horses.
- Identification and prevention of avoidable, unnecessary risks to horses.
- Compliance with governing body regulations and the law.

**FIGURE 1:**
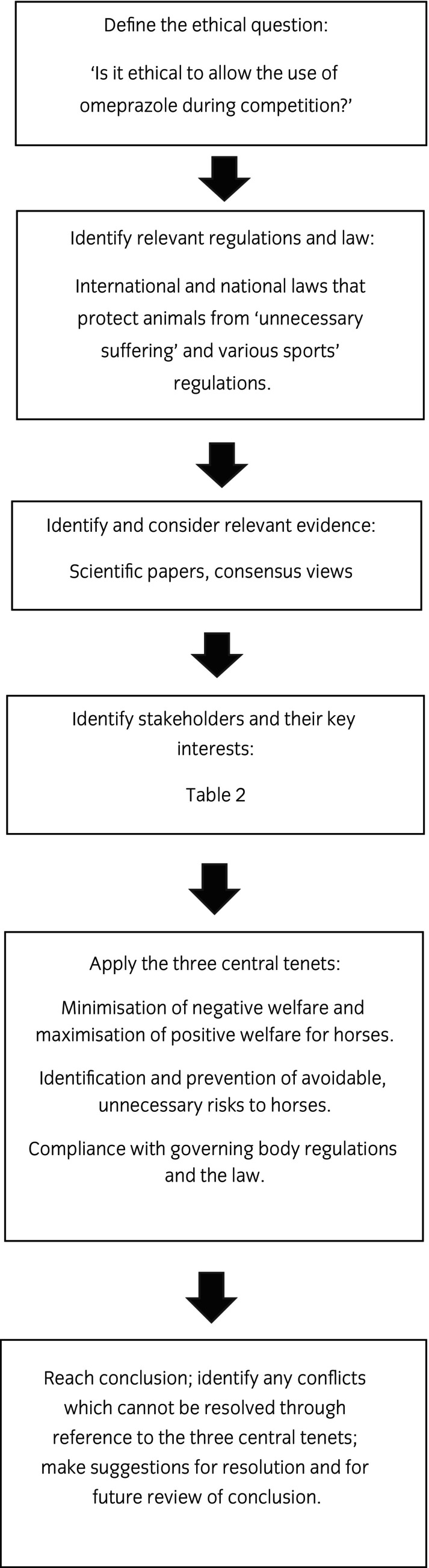
Schematic representation of the application of the steps of Campbell's Ethical Framework for the Use of Horses in Sport to the question: ‘Is it ethical to allow the use of omeprazole during competition?’

The stepwise function of the tool used to consider the question ‘Is it ethical to allow the use of omeprazole during competition?’ is illustrated in Figure 1.

### 2.2 Step 2

The ‘relevant regulations and laws’ were identified as international and national laws that protect animals from ‘unnecessary suffering’ (e.g., the British Animal Welfare Act (2006)) and various sports regulations. The latter are summarised in Table 1.

**TABLE 1:** List of key governing bodies in equestrian sport and their regulations around the use of omeprazole during competition.

Regulator	Rule	Effect of rule
Federation Equestre Internationale	Equine Anti‐Doping & Controlled Medication Regulations 2023 https://inside.fei.org/content/anti‐doping‐rules	Omeprazole allowed during competition
British Horseracing Authority	Prohibited Substances Regulations 2023 https://www.britishhorseracing.com/regulation/anti‐doping‐medication‐control/prohibited‐substances/ Detection time, omeprazole https://www.britishhorseracing.com/wp‐content/uploads/2019/01/Detection‐Time‐for‐omeprazole.pdf	Omeprazole not allowed during competition
Example international regulators of horseracing	E.g., Racing Victoria https://www.racingvictoria.com.au/the‐sport/racing/rules‐of‐racing E.g., International Federation of Horseracing Authorities https://www.ifhaonline.org/	Omeprazole is exempt from prohibition on race days Screening limit stated. No specific statement of required withholding period
Hurlingham Polo Association	Rules and regulations for Polo (2023) https://hpa‐polo.co.uk/download/BB2022‐Rules‐Proof‐4.pdf	Omeprazole allowed during competition
United States Equestrian Federation	USEF Guidelines and Rules for Medications https://www.usef.org/forms‐pubs/2Zp2C_YKs4s/2022‐equine‐drugs‐medications	Omeprazole allowed during competition
Horseracing Integrity and Safety Authority (USA)	Horseracing Integrity and Safety Authority Regulations https://hisaus.org/regulations#controlled	The use of omeprazole is permitted up to 24 h before racing (Rule 4212)
National Reining Horse Association (USA)	National Reining Horse Association Animal Welfare and Medications Policy https://nrha.com/media/pdf/2023/welfare‐meds‐policy.pdf	Omeprazole is a ‘permitted medication’

*Note*: This list is not exhaustive but serves to elucidate the approach of key regulator stakeholders, and differences in those approaches.

### 2.3 Step 3

The application of the framework was informed through background evidence accumulated via a search of PubMed and Web of Science using the search terms: ‘omeprazole AND horses’; reference to allied documents, for example, publication of consensus views, and detailed reference to two recently published articles that address the issue of possible rebound hyperacidity in horses whose omeprazole therapy is discontinued.^13^, ^14^

### 2.4 Step 4

Stakeholders in policy formation/regulation about whether or not the use of omeprazole should be allowed in equestrian sport and their key interests were identified as illustrated in Table 2.

**TABLE 2:** Stakeholders in policy formation, and their key interests.

Stakeholder	Stakeholder's key interests
Horses	Welfare sufficient to result in ‘A good life’ (https://equinewellbeing.fei.org/sf2023.html)
Regulators	Clean sport Protect equine welfare Maintain public confidence in the sport Maintain integrity to the sport for gambling purposes
Riders/trainers	Competitive success Optimise the welfare of the horses who they care for Career satisfaction Business viability
Owners	Competitive success Optimisation of the welfare of the horses who they own Enjoyment of competitions
Equine welfare organisations	Optimisation of equine welfare
‘The public’	Confidence that the welfare of horses being used in sport is properly protected
The gambling public	Confidence that the sport they are betting on is ‘clean’
The veterinary profession	Fulfilment of their responsibility to safeguard the health and welfare of animals under their care. Business financial viability Professional satisfaction and development
Pharmaceutical companies	Medications are generally aimed at promoting equine health and thereby welfare Maximisation of profits (brief withdrawal period unlikely to have a significant impact upon these)
Governments	Having national animal welfare legislation upheld. Receiving tax revenue from gambling

### 2.5 Step 5

The three central tenets were applied to the results of Steps 1–4.

### 2.6 Step 6

A conclusion was reached based on the results of Steps 1–5. Resolution of conflicts through re‐examining the evidence and the harm:benefit analysis was attempted. Suggestions for resolution and future review of the conclusion were made as described in Sections 4 and 5.

## 3 RESULTS

### 3.1 Results of Steps 1–4 of the application of the ethical framework

The peer‐reviewed literature indicated that omeprazole does not improve performance in horses without EGUS^15^ and is an effective treatment for ESGD and less effective for EGGD.^3^, ^16^, ^17^, ^18^, ^19^

However, concerns have been raised as to the potential adverse effects of omeprazole in the horse including rebound gastric hyperacidity that might occur with the discontinuation of treatment.^16^ Treatment of horses with omeprazole causes an increase in serum gastrin (hypergastinaemia) that is evident within 7 days of the initiation of treatment.^20^ The mechanism for this increase is that PPIs increase intra‐gastric pH and thus inhibit negative feedback on gastrin production.^20^ Gastrin regulates gastric acid secretion both through direct effects on the parietal cells of the stomach and via enterochromaffin‐like (ECL) cells in the stomach.^13^, ^21^ In humans, but not demonstrated in horses to date, hypergastrinaemia induced by PPIs also has trophic effects on ECL‐cells further worsening the potential for rebound gastric hyperacidity.

This means that horses treated with PPIs, such as omeprazole, have increased gastric pH due to the drug but simultaneously have hypergastrinaemia that is signalling for increased acid secretion within the stomach. Thus, when treatment with omeprazole is discontinued there is likely to be ‘rebound gastric hyperacidity’ for as long as the hypergastrinaemia (and thus the increased secretion of HCL from parietal cells) persists.^14^ Recent evidence suggests that the hypergastrinaemia induced by omeprazole treatment lasts for only 2–4 days after the discontinuation of treatment in horses that had received 57 days of omeprazole treatment.^12^

Hypergastrinaemia and rebound gastric hyperacidity are likely explanations for the rapid recurrence of ESGD observed in horses when omeprazole is discontinued. Recent research demonstrated a return to pre‐omeprazole treatment prevalence of ESGD when omeprazole was stopped for 3 days (consistent with a ‘2 clear day’ regulatory withholding period), but not when treatment is withheld for only 1 day (equivalent to a ‘day of racing’ regulatory withholding period).^10^

Steps 1–4 inclusive of the application of the ethical framework showed that:

- Whilst stakeholders in equine sports have a variety of (sometimes conflicting) interests, all of them share an interest in optimising equine welfare.
- The incidence of EGUS in horses competing across sports is high.
- Omeprazole is cornerstone of EGUS treatment and the management of associated clinical signs.
- There is currently a discrepancy across equestrian sport in regulation about the use of omeprazole during competitions, with all regulators arguing that their rules best protect equine welfare.
- Recent evidence suggests that withholding omeprazole treatment for two clear days before competition allows recurrence of squamous ESGD, likely due to rebound gastric acidity, whereas withholding treatment on the day of competition only does not have that effect.

### 3.2 Results of Step 5

Based on the results of Steps 1–4, to comply with the central tenets of the ethical framework for the use of horses in sport on (i) minimising negative welfare impacts and optimising positive welfare impacts and (ii) not allowing avoidable, unnecessary suffering, medication should not be withheld from horses being treated with omeprazole for longer than the day of competition.

However, the use of omeprazole within ‘two clear days’ of and on the day of competition is not allowed within the current regulations of some bodies governing horse sport.

### 3.3 Results of Step 6

It is currently not possible for all equestrian sports to fulfil both the welfare‐based first two key considerations of the ethical framework and the third, that is, complying with sports' governing body regulations as some require that omeprazole is withdrawn from longer than 1 day before the competition. This discrepancy cannot be resolved by re‐examining the evidence or the harm:benefit analysis. Indeed, it arises from the fact that recent scientific evidence has demonstrated harms to equine health and welfare associated with persistent hypergastrinaemia and rebound gastric hyperacidity that had not been previously reported in the literature.

## 4 DISCUSSION

In human sport, decisions around whether to allow the use of drugs during competition are commonly presented in a ‘justice’ ethical framework^7^ with the emphasis (whilst acknowledging that complete equality of opportunity is impossible) being on preventing one athlete having an unfair advantage over others. Similar sentiments are expressed by the main regulators of equine sport.^22^ The FEI website, for example, states that the creation of ‘A universal and level playing field’ is one of its core values, whilst the website of the British Horseracing Authority (BHA) claims that part of the BHA's role is to ‘encourage (…) the honest majority to do the right thing, and prevent the dishonest minority from gaining an unfair advantage, thus ensuring a level playing field for all’.

The principle of justice, however, is not sufficient to answer the question of whether the use of omeprazole should be allowed in equestrian competition, since the requirement for a ‘level playing field’ in this specific context could be satisfied either by not allowing the use of omeprazole at all, or by allowing its use in all horses. This is explained as follows: a blanket ban on the use of omeprazole during competition theoretically ensures that no one horse has an omeprazole‐derived advantage over another, and thus satisfies the principle of justice. However, the principle of justice could equally be satisfied by allowing the use of omeprazole in all horses because omeprazole functions to reduce EGUS and its associated clinical signs (including poor performance) and negative welfare effects but not, so far as is proven, to enhance athletic performance above that might be expected in the same horse where it was healthy (i.e., not suffering from EGUS). It, therefore, follows that if there were a blanket permission to use omeprazole during competition then all horses (i.e. horses suffering from EGUS and treated with omeprazole, any horses that were for some reason treated but did not have EGUS, and healthy non‐treated horses) could be considered to be competing at an equally ‘normal’ level of health, thus fulfilling the requirement for justice.

The principle of justice is additionally insufficient to answer the question of whether the use of omeprazole should be used in equestrian competition because it fails to take into account the vulnerability of equine athletes. It is recognised that the consent of human athletes to compete can be influenced by external pressures,^23^ and that it is thus an over‐simplification to state that human athletes consent to participate in sport and equine athletes do not. Nonetheless, the second part of that statement, that is, that equine athletes are unable to give informed consent to involvement in equestrian competition is undeniably true. Equine athletes are in a vulnerable position and, whether for deontological, Virtue Ethics or utilitarian reasons,^24^ it is incumbent upon the humans who use them in sport to optimise their welfare.

Those involved in equestrian sport, therefore, need a method of ethical analysis that prioritises equine welfare above all else, because that is ‘the right thing to do’^25^ and will thereby help to maintain the social licence to use horses in sport. The use of the Ethical Framework for the use of Horses in Sport provides such a method by combining an essentially utilitarian analysis with a deontological requirement to fulfil its three established key considerations.

The authors' application of Campbell's Ethical Framework for the use of Horses in Sport to the question ‘Should the use of omeprazole be allowed during equestrian competition?’ showed that all stakeholders share an interest in optimising equine welfare. It also confirmed that there are current regulatory discrepancies around the question.

The review of the scientific and allied literature that provided the evidence base for the analysis indicated that:

- EGUS occurs commonly in horses involved in competitive sport
- Omeprazole is a variably effective treatment for EGUS and alleviates clinical signs that cause poor welfare and
- Withholding omeprazole treatment for longer than the day of competition causes a deterioration in disease status. This can be expected to result in an exacerbation of clinical signs and thus a deterioration in welfare status
- In humans discomfort due to the rebound exacerbation of gastric acidity that accompanies hypergastrinaemia at the time of abrupt omeprazole withdrawal occurs rapidly.^20^ To the best of our knowledge, it is reasonable to expect a similarly rapid recurrence of adverse clinical symptoms and therefore physical discomfort and a negative welfare state in horses whose omeprazole treatment is withdrawn for 1 day, even in the absence of the recurrence of lesions within that timeframe. Therefore, whilst that expectation remains to be proven through behavioural studies, the precautionary principle should be followed: if horses are being treated with omeprazole outside of competition then treatment on the day of competition should be permitted on welfare grounds

## 5 CONCLUSIONS

Our analysis thus leads us to the following conclusions about whether the use of omeprazole should be allowed during competition:

1. Given the insufficiencies (described above) of the principle of justice as a method of approaching this question, and given that the use of omeprazole does not anyway enhance performance above ‘normal healthy’ levels, ethical decision‐making about whether or not to allow the use of omeprazole during equestrian competition should rely primarily upon the scientific evidence base about health and welfare effects. Such evidence informs the application of the Ethical Framework for the Use of Horses in Sport.
  The scientific evidence shows that EGUS recurs if omeprazole treatment is withdrawn ‘2 clear days’ before competition. Based on that scientific evidence withdrawal should be for not longer than 1 day. There is no scientific evidence that omeprazole has an enhancing effect on performance, and therefore no ‘level field of play’ argument against permitting the use of omeprazole during competition. It is highly likely—although it remains to be proven through behavioural studies—that horses experience physical discomfort associated with acid rebound when treatment is withdrawn for even 1 day, even if EGUS lesions do not recur during that timeframe. Following the precautionary principle, therefore, if horses are being treated with omeprazole outside of competition then treatment on the day of competition should be permitted on welfare grounds.
2. This recommendation (1) is consistent with fulfilment of the first two, welfare‐based key considerations of the ethical framework. However, the third key consideration, that is, complying with governing body regulations cannot currently be reconciled with our recommendation for all equine sports since some require that omeprazole be withheld during competition. Revision of regulations around the use of omeprazole during competition by those governing bodies is, therefore, necessary to safeguard the ethical use of horses in sport.

However, there are some caveats to these conclusions. All regulators currently allow the use of omeprazole outside of competition. In humans, in addition to hypergastrinaemia, the use of omeprazole can cause such adverse effects as increased fracture risk, increased risk of antimicrobial‐associated diarrhoea, kidney injury and hypomagnesia.^26^ To date, these adverse effects have not been demonstrated in horses. A detailed discussion of the possible side effects of omeprazole treatment in horses including effects on calcium digestibility and microbiota is beyond the scope of this article but is reviewed in Sykes.^16^ Whilst it can be concluded that withholding omeprazole for longer than 1 day in horses that are routinely being treated with it is ethically undesirable for welfare reasons, the authors have confined our analysis to the question of whether the use of omeprazole should be allowed during competition and have not said anything about possible health and thus welfare effects of the out‐of‐competition treatment with omeprazole that all regulators of equine sport currently allow.

Furthermore, given the high incidence of EGUS in competition horses^3^ there remains an underlying ethical issue about whether humans should be subjecting horses to management, training, and competition regimens that result in poor health and welfare. That broader topic is also outside the scope of this article, other than to comment that evidence on how the incidence of EGUS in competition horses compares to that in leisure horses should be used to inform welfare‐based decisions about how competition horses should be managed.

## FUNDING INFORMATION

World Horse Welfare.

## CONFLICT OF INTEREST STATEMENT

Madeleine L. H. Campbell declares no conflict of interest. Benjamin W. Sykes has active consultancies with Kelato and Mayo Health, who have products in the EGUS space. He has also, within the past 3 years, worked, for, or received funding from, the following companies with commercial interests in the EGUS space: Abbey Laboratories, A‐Vet, Equestra Australia, Health Food Symmetry, Hong Kong Jockey Club, Norbrook UK, Salfarm Denmark, and Troy Australia. None of the aforementioned companies had any input into this manuscript.

## AUTHOR CONTRIBUTIONS

**Madeleine L. H. Campbell:** Conceptualization; writing – original draft; methodology; writing – review and editing. **Benjamin W. Sykes:** Methodology; writing – original draft; writing – review and editing.

## DATA INTEGRITY STATEMENT

No new data were created or analysed in this study.

## ETHICAL ANIMAL RESEARCH

Not applicable.

## INFORMED CONSENT

Not applicable.

## Data Availability

Data sharing is not applicable to this article as no new data were created or analysed in this study.
